# Systems Biology of Gibberellin Induced Plant Cell Growth

**DOI:** 10.3389/fpls.2012.00173

**Published:** 2012-08-02

**Authors:** Wagner L. Araújo, Alisdair R. Fernie

**Affiliations:** ^1^Departamento de Biologia Vegetal, Universidade Federal de ViçosaViçosa, Minas Gerais, Brazil; ^2^Max-Planck-Institut für Molekular PflanzenphysiologiePotsdam – Golm, Germany

By analogy to their animal counterparts, plant hormones were originally recognized as regulators of growth and development (van Overbeek, 1966; Galston and Davies, 1969; Santner and Estelle, 2009). They have subsequently been demonstrated to achieve this by modulating both processes in response to both intrinsic and environmental cues (Wolters and Jurgens, 2009). Accordingly it is currently accepted that hormones not only exert intrinsic growth control but also mediate adaptation of plant development to transiently changing conditions (Wolters and Jurgens, 2009). Equally accepted is the fact that they play key roles in the regulation of immune responses to microbial pathogens, insect herbivores, and beneficial microbes (Pieterse et al., 2012). In brief the classical developmental growth regulators are considered as auxin, brassinosteroid, cytokinin, and gibberellin (GA), whilst abscisic acid, ethylene, jasmonic, and salicylic acid are often implicated in stress responses (Browse, 2009; Vlot et al., 2009; Wolters and Jurgens, 2009). However, the very recent identification of strigolactones as a further developmental growth regulators (Gomez-Roldan et al., 2008; Umehara et al., 2008; Alder et al., 2012) suggests that our current list of phytohormones may not yet be complete.

Growth hormone studies over the past 20 years have been dominated by the analysis of mutants and transgenic plants. These studies provided us with extensive “parts lists” of genes involved in hormone synthesis and catabolism or encoding receptors and signaling components for most classical hormones (Hedden and Phillips, 2000; Hedden, 2003; Yamaguchi, 2008; Wolters and Jurgens, 2009). However, although many components of the plant hormone signaling network have been identified in this way, less consideration has been given to understanding the precise mechanisms by which hormones can regulate plant growth and development *per se*. Two recent elegant studies have integrated experimental data and multi-scale mathematical modeling approaches in order to explore the interplay between GAs signaling and the root elongation zone (Band et al., 2012) and to investigate the role of the various feedback loops in GA signaling (Middleton et al., 2012). In these ground-breaking studies the authors utilize experimental data to validate their model by comparing predicted mRNA levels with that obtained from transcriptomics. As such they not only provide important fundamental insight into hormonally regulated plant growth but also serve as powerful examples of the marriage of experimental and theoretical approaches in plant systems biology.

Understanding the development of root systems is vital in efforts toward maintaining/improving food security. Roots both provide anchorage and facilitate acquisition of water and nutrients from the soil with growing roots exploring their local environments in order to exploit these resources (Lynch, 1995). The understanding of the regulation of developmental and physiological process underlying plant growth is of particular importance since greater growth will lead to a larger leaf area which may ultimately compound final gains in productivity. Thus in order to explore the relationship between GAs and root growth Band et al. (2012) used a multi-scale mathematical model associated with measurements of root growth dynamics and investigated the distribution of GA within the root elongation zone. A significant gradient in GA levels was simulated in the model based on the fact that rapid cell expansion is associated with an expected dilution of GA. The incorporation of the GA signaling network allowed an *in silico* simulation of how GA would affect downstream components including the growth-repressing DELLA proteins. It was also possible to predict a gradient in DELLA proteins which provides a reasonable explanation for the observation of a reduction in growth exhibited in cells close to the end of the elongation zone (Band et al., 2012). The authors also simulated systems in which GA levels were reduced by genetic or pharmacological approaches. Accordingly by modeling these scenarios it was possible to predict how dilution-induced spatial variations in DELLA concentration can explain the growth dynamics of *Arabidopsis* root cells (Band et al., 2012). In a similar vein a recent purely experimentally based comparison of molecular changes in transcript and metabolite levels demonstrated that a low GA level mainly affects growth by uncoupling growth from carbon availability (Ribeiro et al., 2012).

It is now more than 50 years ago that GA was first proposed to act as an inhibitor of an inhibitor of growth (Stowe and Yamaki, 1959), a concept reinforced by the recently identified molecular mechanisms of GA action (Davière et al., 2008; Yamaguchi, 2008; Achard and Genschik, 2009). The GA-responsive inhibitors of plant growth proposed by Brian (1957) are now known as DELLA proteins (usually referred to as DELLAs). DELLAs are a subfamily of the GRAS family of putative transcriptional regulators and are named for a conserved set of N-terminal amino acids (Asp-Glu-Leu-Leu-Ala; Bolle, 2004). It is well known that GA binds its receptor, GID1, to form a complex that mediates the degradation of DELLAs (Hedden and Thomas, 2012). By this means, GAs relieves DELLA-dependent growth repression and therefore control plant growth by regulating the degradation of growth-repressing DELLA proteins (Sun, 2011; Hedden and Thomas, 2012). It is noteworthy that GA also regulates the expression of *GID1*, *GA20ox*, and *GA3ox*, and there is also evidence that it regulates *DELLA* expression itself (Hedden and Thomas, 2012). In summary it is clear that the concentration of biologically active GAs at their site of action is tightly regulated being additionally modulated by numerous developmental and environmental cues (Hedden and Thomas, 2012). Bearing this in mind, in their companion study to the paper by Band et al. (2012), Middleton et al. (2012) integrated mathematical modeling and experimental analysis in an attempt to understand how these feedback loops are able to interact and thereby control GA signaling. The model simulations presented revealed exceptionally good agreement with *in vitro* data concerning signal transduction and the underlying biosynthetic pathways as well as *in vivo* data concerning the expression levels of GA-responsive genes. In this study they identified that GA-GID1 interactions were observed in two independent timescales. Furthermore, their model accurately predicts the response to exogenous GA following a range of chemical and genetic perturbations and suggests that the regulation of *GA20ox* transcription plays a significant role in both modulating the level of endogenous GA and generating overshoots after the removal of exogenous GA.

In recent years much effort has been expended on elucidating the physiological functions of the various genes regulated by GA (Yamaguchi, 2008). By contrast, directed studies concerning the effect of GA on gene expression, energy metabolism, and growth are rare. That said, studies of characterization of GA-regulated genes have provided interesting insights, for example, the regulation of the pyruvate dehydrogenase kinase 1 (PDK1) by GA. Such studies have showed that GA modulates the activity of mitochondrial pyruvate dehydrogenase by regulating *PDK1* expression and subsequently controlling growth of rice plant (Yazaki et al., 2003; Jan et al., 2006). These results suggest that GA might be able to modify primary metabolism at the entry point of tricarboxylic acid cycle (TCA). Furthermore, several studies surveying overexpression of genes associated with GA biosynthesis or catabolism have indicated the important roles of GA levels on the setting of transcriptional programs influencing plant growth (Biemelt et al., 2004; Dayan et al., 2010). It will likely prove important in the future to additionally consider metabolic influences on the levels of GA. For example roots of plants harboring reduced activities of TCA cycle enzymes displayed modified GA levels and therefore modified metabolism (Van der Merwe et al., 2009) whilst tomato plants with reduced levels of the TCA cycle enzyme 2-oxoglutarate dehydrogenase were recently characterized by early leaf senescence and a modified fruit ripening due to differences in the levels of bioactive GA (Araújo et al., 2012).

Although the studies highlighted above have clearly enhanced our understanding of the effect of GA in specific developmental process, they provide little information concerning the general role of GA in the regulation of plant metabolism and growth. It will thus likely be highly informative to include metabolic aspects into future expanded versions of these models. The future development of plant systems biology will ultimately require capacities at both theoretical and experimental levels to develop both independently and in concert (Fernie, 2012), and progress in these areas as they relate to research on plant hormones and their role in their regulation of plant growth and development will certainly maintain a prominent role in this emerging field for many years to come. These two fantastic papers provide valuable blueprints of the current state-of-the-art.

*In summary* it seems that plant systems biology is here to stay just as much as plant molecular biology. The combination of synergistic application and further development of quantitative experimentation, modeling and theory as discussed above is a promising approach that will bring not only plant biology but biology in general to the next systems level. Accordingly the ultimate challenge of genomics, transcriptomics, proteomics, metabolomics, will be moving to the characterization of not only but also single molecules and single cells. This will clearly require rapid global analyses with high data quality and low cost per informational unit analyzed.
